# Multicentre analysis of second-line antiretroviral treatment in HIV-infected children: adolescents at high risk of failure

**DOI:** 10.7448/IAS.20.1.21930

**Published:** 2017-09-13

**Authors:** Ragna S. Boerma, Torsak Bunupuradah, Dorothy Dow, Joseph Fokam, Azar Kariminia, Dara Lehman, Cissy Kityo, Victor Musiime, Paul Palumbo, Annelot Schoffelen, Sam Sophan, Brian Zanoni, Tobias F. Rinke de Wit, Job C.J. Calis, Kim C.E. Sigaloff, for the Paediatric Second-line Study Group

**Affiliations:** ^a^ Amsterdam Institute for Global Health and Development and Department of Global Health, Academic Medical Center of the University of Amsterdam, Amsterdam, The Netherlands; ^b^ Global Child Health Group, Emma Children’s Hospital, Academic Medical Center of the University of Amsterdam, Amsterdam, The Netherlands; ^c^ HIV-NAT, The Thai Red Cross AIDS Research Centre, Bangkok, Thailand; ^d^ Department of Pediatrics, Division of Infectious Diseases, Duke University Medical Center, Durham, NC, USA; ^e^ Chantal BIYA International Reference Centre for Research on HIV/AIDS Prevention and Management, Yaoundé, Cameroon; ^f^ Faculty of Medicine and Biomedical Sciences, University of Yaoundé I, Yaoundé, Cameroon; ^g^ Faculty of Medicine and Surgery, University of Rome Tor Vergata, Rome, Italy; ^h^ The Kirby Institute, UNSW Australia, Sydney, Australia; ^i^ Division of Human Biology, Fred Hutchinson Cancer Research Center and the Department of Global Health, University of Washington, Seattle, WA, USA; ^j^ Joint Clinical Research Centre, Kampala, Uganda; ^k^ Department of Paediatrics and Child Health, College of Health Sciences, Makerere University, Kampala, Uganda; ^l^ Section of Infectious Diseases and International Health, Geisel School of Medicine at Dartmouth, Lebanon, NH, USA; ^m^ Department of Internal Medicine and Infectious Diseases, University Medical Center Utrecht, Utrecht, The Netherlands; ^n^ Ndlovu Research Consortium, Julius Center for Health Sciences and Primary Care, University Medical Center Utrecht, Utrecht, The Netherlands; ^o^ Child Health Improvement Clinic, Department of HIV/AIDS-TB, National Pediatric Hospital, Phnom Penh, Cambodia; ^p^ Massachusetts General Hospital, Division of Infectious Diseases, Boston, MA, USA; ^q^ Emma Children’s Hospital, Department of Pediatric Intensive Care, Academic Medical Center of the University of Amsterdam, Amsterdam, The Netherlands; ^r^ Department of Infectious Diseases, Division of Internal Medicine, Leiden University Medical Center, Leiden, The Netherlands

**Keywords:** antiretroviral treatment, children, adolescents, virologic failure, HIV-1, second-line treatment

## Abstract

**Introduction**: The number of HIV-infected children and adolescents requiring second-line antiretroviral treatment (ART) is increasing in low- and middle-income countries (LMIC). However, the effectiveness of paediatric second-line ART and potential risk factors for virologic failure are poorly characterized. We performed an aggregate analysis of second-line ART outcomes for children and assessed the need for paediatric third-line ART.

**Methods**: We performed a multicentre analysis by systematically reviewing the literature to identify cohorts of children and adolescents receiving second-line ART in LMIC, contacting the corresponding study groups and including patient-level data on virologic and clinical outcomes. Kaplan–Meier survival estimates and Cox proportional hazard models were used to describe cumulative rates and predictors of virologic failure. Virologic failure was defined as two consecutive viral load measurements >1000 copies/ml after at least six months of second-line treatment.

**Results**: We included 12 cohorts representing 928 children on second-line protease inhibitor (PI)-based ART in 14 countries in Asia and sub-Saharan Africa. After 24 months, 16.4% (95% confidence interval (CI): 13.9–19.4) of children experienced virologic failure. Adolescents (10–18 years) had failure rates of 14.5 (95% CI 11.9–17.6) per 100 person-years compared to 4.5 (95% CI 3.4–5.8) for younger children (3–9 years). Risk factors for virologic failure were adolescence (adjusted hazard ratio [aHR] 3.93, *p* < 0.001) and short duration of first-line ART before treatment switch (aHR 0.64 and 0.53, *p* = 0.008, for 24–48 months and >48 months, respectively, compared to <24 months).

**Conclusions**: In LMIC, paediatric PI-based second-line ART was associated with relatively low virologic failure rates. However, adolescents showed exceptionally poor virologic outcomes in LMIC, and optimizing their HIV care requires urgent attention. In addition, 16% of children and adolescents failed PI-based treatment and will require integrase inhibitors to construct salvage regimens. These drugs are currently not available in LMIC.

## Introduction

Worldwide, the number of HIV-infected children receiving antiretroviral treatment (ART) has more than doubled since 2010 to an estimated 823,000 in 2014 [1]. In low- and middle-income countries (LMIC), 97% of children on ART are on first-line treatment, and only 3% receives second-line ART [2]. However, with the increasing paediatric ART coverage in LMIC [1], the number of children failing first-line and requiring second-line options will rise.

Currently, the World Health Organization (WHO) recommends that HIV-infected children three years or older of age start non-nucleoside reverse-transcriptase inhibitor (NNRTI)-based ART as a first-line regimen and to switch to a protease inhibitor (PI)-based second-line regimen in case of treatment failure. Children younger than three years are advised to start PI-based first-line ART [3]. As PI-based treatment is costly and logistically challenging, local clinics might still prefer to start an NNRTI-based regimen as first-line ART in young children. Moreover, due to late diagnosis and linkage to care, perinatally HIV-infected children may only start ART after the age of three years [4–6]. As a consequence, 56% of children in LMIC still receive a nevirapine-based first-line regimen and switch to PI-based ART in case of failure [7]. Children have higher rates of first-line ART failure compared to adults [8–11]. The population of HIV-infected adolescents is growing as perinatally infected children now survive until adulthood, and HIV treatment in adolescents is especially challenging [12–14].

In order to ensure adequate long-term treatment for children and adolescents, evaluation of current second-line treatment outcomes is essential. Data, however, are scarce [15], and the available studies have small sample sizes, use different definitions of virologic failure and have different follow-up periods [16–19]. Therefore, it is hard to compare results across different cohorts. In adults, multicentre studies and meta-analyses of second-line ART have been conducted [15,20], but such analyses are missing for children. We performed a multicentre analysis of children and adolescents in LMIC to assess second-line treatment outcomes, describing the rate of virologic failure and identifying its predictors. In this way, we evaluated the effectiveness of second-line ART and estimated the need for third-line regimens.

## Methods

We conducted a systematic review of the literature according to PRISMA guidelines [21] to identify cohorts of children receiving second-line ART in LMIC. We systematically searched the literature in Medline through PubMed, Embase, the Literatura Latino Americana de Ciencias de Salud and the African Index Medicus, as well as conference abstracts of the International AIDS Society, the Conference on Retroviruses and Opportunistic Infections and the HIV Pediatric Workshops of 2014 and 2015 to identify second-line cohorts whose results had not yet been published. The complete search strategy for published articles is provided in Supplementary Table S1. For conference abstracts, we used the search term “second-line” or “treatment-experienced”.

### Study selection

We searched for cohort studies, cross-sectional studies and randomized controlled trials reporting second-line virologic treatment outcomes of children and adolescents living in LMIC (according to the World Bank 2016 definition [22]). Studies were eligible if they reported on at least five children on a PI-based second-line regimen. Within each eligible study, we further excluded: children aged <3 years or >18 years at switch to second-line ART; children who had received less than six months of first-line ART before switching; children who had received a first-line regimen not containing an NNRTI; children having either a first- or a second-line regimen consisting of dual therapy or monotherapy; and children without any viral load (VL) results during the first five years of second-line ART. In order to create a homogeneous group of participants, we selected only children on ritonavir-boosted lopinavir (LPV/r)-based ART, reflecting the 2013 WHO guidelines on paediatric second-line ART [23]. Because of the various changes in treatment guidelines for children younger than three years (from NNRTI-based first-line and PI-based second-line in 2010 to PI-based first-line and raltegravir-based second-line in 2016 [3,23,24]), we included only participants older than three years of age.

Studies were selected independently by two reviewers, and any disagreement was resolved by discussion between both. The authors of eligible articles were approached by email with a request to share the patient-level data of their cohort. If the study group agreed to share data, a data-sharing agreement was signed by both parties. Anonymized data sets were shared by email and were collected in one central database. If different study groups had included participants from the same study sites, we checked with the corresponding authors that none of the participants were included in our data set twice. Only the first author had direct access to all data in order to maintain confidentiality.

### Data management

Data sets were provided by the corresponding authors in Stata®, SPSS® or Excel® format. The following variables were requested: sex; age at switch; calendar year at switch; country of residence; duration of first-line ART; drugs used in first-line regimen; reason for switching to second-line ART; drugs used in second-line regimen; VL, CD4 count/CD4 percentage and WHO/CDC (Centers for Disease Control and Prevention) HIV stage at switch; VL during follow-up and period between switch and VL measurement; status at moment of censoring (in care, dead, lost to follow-up, transferred out); and date of end of study. In order to compare CD4 count and CD4 percentages in different age groups, we constructed a variable, called CD4 status, defined as: normal - CD4 count >500 (for children five years or older) or CD4% >25 (for children younger than five years); diminished, CD4 count 100–500 (for children five years or older) or CD4% 10–25% (for children less than five years); and immunodeficient, CD4 count <100 (for children five years or older) or CD4% <10% (for children younger than five years).

### Risk of bias assessment

To assess the potential risk of bias in each cohort, we developed a list of five criteria relevant for this analysis, based on existing checklists of the Newcastle Ottowa Scale [25], the Strengthening the Reporting of Observational studies in Epidemiology (STROBE) [26] and the Cochrane Collaboration tool for assessing the risk of bias [27]. For each criterion, a study could score 1 if the criterion was met and 0 if the criterion was not met or not reported. Each cohort could obtain a maximum score of 5. Cohorts with a score of ≥4 were considered to have a lower risk of bias and below 4 a higher risk. Criteria and scores of each study are provided in Supplementary Table S2.

### Data analysis

We defined young children as individuals aged nine years or younger and adolescents as individuals aged 10–18 years at switch. Baseline characteristics of young children and adolescents were described using counts and percentages or medians and interquartile ranges (IQRs). We did not use multiple imputation to adjust for missing data if these data were missing at the cohort level. For categorical variables, we added a “missing” category for individuals with missing data for that variable. Kaplan–Meier survival analysis was used to assess the cumulative incidence of virologic failure overall and separately for younger children and adolescents. Difference in virologic failure rates between groups was assessed using a log-rank test. In the primary analysis, we defined virologic failure according to the WHO definition of two consecutive VL measurements >1000 copies/ml after at least six months of second-line ART [3]. As not all studies used a second confirmatory VL measurement to define failure, we conducted a secondary analysis in which we defined failure as a single VL >1000 copies/ml after at least six months of second-line ART. Associations between virologic failure and age, sex, time spent on first-line ART, calendar year of switch and VL and CD4 count/percentage at switch were assessed using a Cox proportional hazard model, clustered by study cohort and by number of VL results per child (<5 results, 5–15 results and >15 results). Variables associated with failure at *p* < 0.10 in the univariable analysis and clinically relevant variables were added to the multivariable model. We checked for collinearity by calculating the variance inflation factor. The proportional hazard assumption was tested by calculating Schoenfeld residuals. To test for the robustness of our data, we conducted three sensitivity analyses in which we only included children in sub-Saharan Africa, only children with known VL and CD4 count/percentage results at switch to second-line ART or only studies with a perceived lower risk of bias. A two-sided *p*-value of ≤0.05 was considered significant. Data were analysed using Stata 12® (StataCorp LP, College Station, TX, USA).

### Ethics

All included study cohorts obtained ethical clearance from their local ethical committee. All studies have been conducted in compliance with Good Clinical Practice guidelines and the principles of the Declaration of Helsinki. All participants in the included study cohorts provided informed consent to participate in the corresponding studies.

## Results

### Search

**Figure 1.**
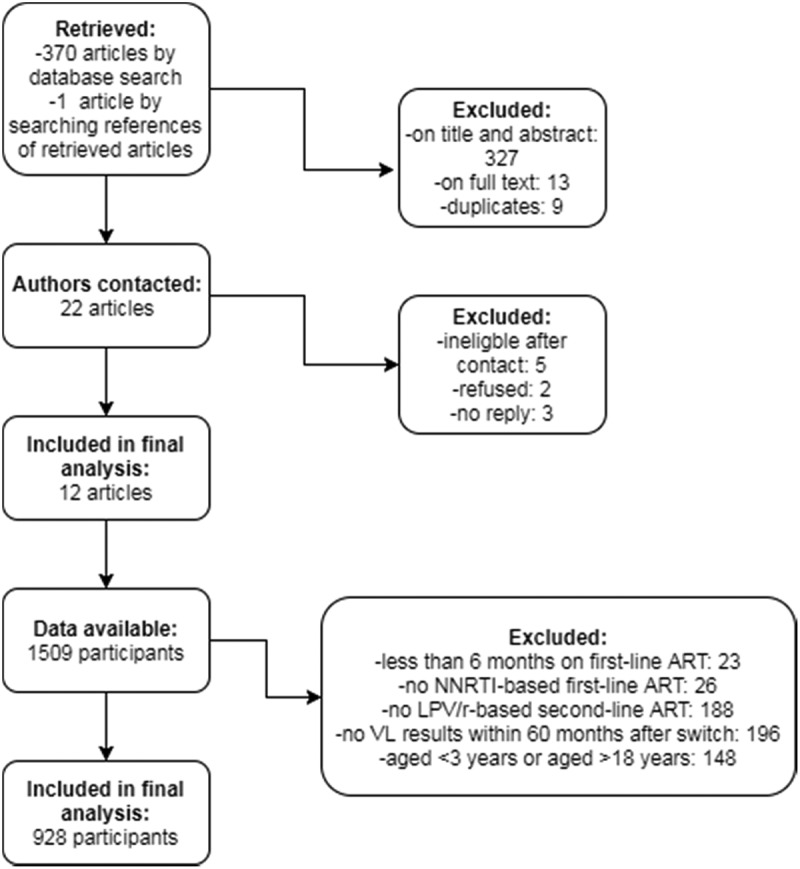
Flow chart of study and participant selection. NNRTI: non-nucleoside reverse-transcriptase inhibitor; LPV/r: ritonavir-boosted lopinavir; VL: viral load.

Our search strategy retrieved a total of 370 articles of which 340 articles were excluded. Of 371 retrieved conference abstracts, 367 abstracts were excluded. Main reasons for exclusion were not concerning second-line treatment, inclusion of adults rather than children or not reporting virologic treatment outcomes. Thirty articles and four abstracts were eligible for inclusion. The four abstracts all represented published articles, and these articles were already part of the 30 articles retrieved by our literature search. After full text screening of the remaining articles, 21 authors were contacted to participate which resulted in 12 contributing data sets (Figure 1).

### Characteristics of included studies

The 12 remaining cohorts represent 928 children receiving LPV/r-based second-line ART in 14 countries in Asia and sub-Saharan Africa. Characteristics of children included are shown in Table 1, and study characteristics are described in Supplementary Table S3. Median age at switch was 9.0 years (IQR 6.1–12.0). Fifty-six children (6.0%) were enrolled in a randomized controlled trial, and all other children were included in observational cohort studies. Median time on first-line ART prior to switch was 36.0 months (IQR 21.6–51.6). The most common first-line regimen was zidovudine/lamivudine/nevirapine (AZT/3TC/NVP) in 235 children (25.8%), and the most common second-line regimen was AZT/3TC/LPV/r in 194 children (21.3%). A median of three VL results (IQR 2–6) per individual were reported during follow-up (for children: 3 (IQR 2–6), for adolescents: 4 (IQR 2–7)). At the time of censoring, 717 (77.3%) children were in care and on treatment, 20 (2.2%) were lost to follow-up, 24 (2.6%) had died, 72 (7.8%) had transferred out and for 95 (10.2%) children the status at end of follow-up was not recorded. Of all young children, 436/480 (90.8%) were still in care and on treatment at censoring compared to 281/353 (79.6%) of adolescents, *p* < 0.001 (χ^2^ test).

**Table 1.** Characteristics of children and adolescents included in the multicentre analysis

		Children (3–9 years old)		Adolescents (10–18 years old)		Total	
		*N*	%	*N*	%	*N*	%
Total		532	100	396	100	928	100
Region	Sub-Saharan Africa	220	41.4	160	40.4	380	40.9
	Asia	312	58.6	236	59.6	548	59.1
Calendar year of treatment switch	2003–2007	52	9.8	18	4.5	70	7.5
	2008–2010	369	69.4	278	70.2	647	69.7
	2011–2014	111	20.9	100	25.3	211	22.7
Gender	Boys	307	57.7	185	46.7	492	53.0
	Girls	225	42.3	211	53.3	436	47.0
Viral load at switch (copies/ml)	<1000	15	2.8	22	5.6	37	4.0
	1000–10,000	74	13.9	52	13.1	126	13.6
	10,000–100,000	125	23.5	90	22.7	215	23.2
	>100,000	121	22.7	58	14.6	179	19.3
	Not available	197	37.0	174	43.9	371	40.0
CD4 status at switch^a^	Normal	129	24.2	51	12.9	180	19.4
	Diminished	122	22.9	117	29.5	239	25.8
	Immunodeficient	44	8.3	52	13.1	96	10.3
	Not available	237	44.5	176	44.4	413	44.5
Duration of first-line antiretroviral treatment	<24 months	190	35.7	75	18.9	265	28.6
	24–48 months	219	41.2	158	39.9	377	40.6
	>48 months	122	22.9	162	40.9	284	30.6
	Not available	1	0.2	1	0.3	2	0.2

^a^Normal: CD4 count >500 or CD4% >25; diminished: CD4 count 100–500 or CD4% 10–25%; immunodeficient: CD4 count <100 or CD4% <10%. Status is based on CD4% for children younger than five years and on CD4 count for children five years or older.

### Virologic failure

**Figure 2.**
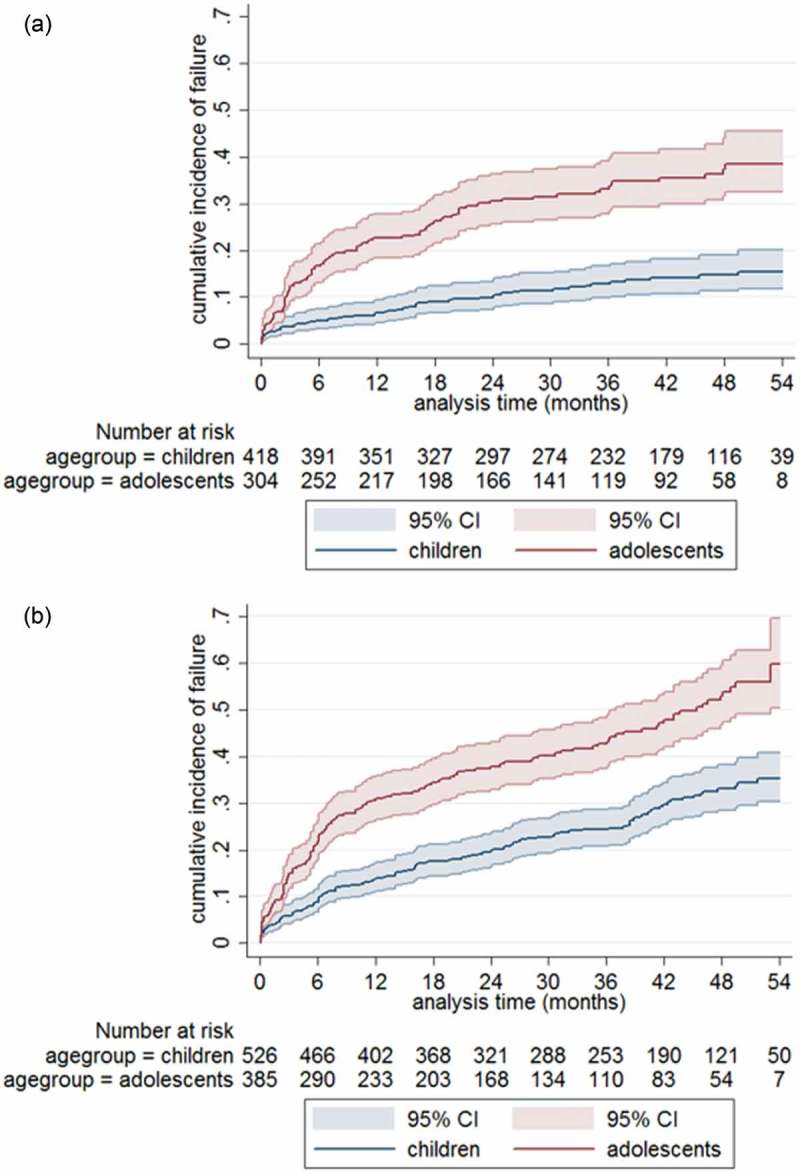
(a, b) Cumulative incidence of virologic failure among children and adolescents on second-line treatment. (a) Primary analysis and (b) secondary analysis. Virologic failure in the primary analysis is defined as two consecutive VL results >1000 copies/ml after at least six months of second-line treatment (*n* = 722) and in the secondary analysis as a single VL >1000 copies/ml after at least six months of second-line treatment (*n* = 911). T0 is set at six months after treatment switch; total follow-up time is 60 months.

Of the 928 children on second-line PI-based ART, 722 (77.8%) children had at least two VL results during follow-up, were still in care and on treatment after six months of second-line ART and were included in the primary analysis (virologic failure defined as two consecutive VL >1000 copies/ml). Over a total follow-up time of 1882 person-years, 154 children experienced virologic failure, resulting in a failure rate of 8.2 (95% CI 7.0–9.6) per 100 person-years. After 12, 24 and 60 months, failure rates were 10.0% (95% CI 8.0–12.5), 16.4% (95% CI 13.9–19.4) and 25.3% (95% CI 21.7–29.2), respectively. In children, the failure rate was 4.5 (95% CI 3.4–5.8) and in adolescents 14.5 (95% CI 11.9–17.6) per 100 person-years. After 24 months, 9.1% (95% CI 6.6–12.4) of younger children and 26.3% (95% CI 21.7–31.8) of adolescents had experienced virologic failure, *p* < 0.001 (Figure 2(a)). For children from sub-Saharan Africa, the failure rate was 6.7 (95% CI 4.9–9.1) per 100 person-years and for children from Asia 8.9 (95% CI 7.4–10.6) per 100 persons-years.

Out of 928 children, 911 (98.2%) children had at least one VL result, were still in care and on treatment after six months of second-line ART and were included in the secondary analysis (virologic failure defined as a single VL >1000 copies/ml). Over a total follow-up time of 2058 person-years, 307 children experienced virologic failure, resulting in a failure rate of 14.9 (95% CI 13.3–16.7) per 100 person-years. After 24 months, 17.5% (95% CI 14.4–21.2) of young children and 34.4% (95% CI 29.7–39.5) of adolescents failed, *p* < 0.001 (Figure 2(b)).

### Predictors of virologic failure

In a multivariable analysis, adolescents had a higher risk of virologic failure compared to younger children (adjusted hazard ratio (aHR) 3.93 (95% CI 2.67–5.78), *p* < 0.001). Children who had spent more time on first-line ART had a lower risk: compared to <24 months of first-line ART, the aHR was 0.64 (95% CI 0.42–0.96), *p* = 0.032, for those on first-line ART for 24–48 months, and 0.53 (95% CI 0.33–0.85), *p* = 0.008, for those on first-line ART for >48 months. Sex, calendar year of switch, VL and CD4 status at switch were not associated with virologic failure (Table 2). Adolescence was the only factor associated with virologic failure in three sensitivity analyses, with an aHR of 3.08 (95% CI 1.35–7.02, *p* = 0.007) for children in sub-Saharan Africa only, an aHR of 5.11 (95% CI 2.66–9.82, *p* < 0.001) for children with a known VL and CD4 status at switch and an aHR of 4.32 (95% CI 1.69–11.04, *p* = 0.002) for children in studies with a perceived lower risk of bias (Supplementary Table S4).

**Table 2.** Factors associated with second-line virologic failure

			Univariable model		Multivariable model	
		Failure rate per 100 person-years (95% CI)	Hazard ratio	*p*	Adjusted hazard ratio	*p*
Age group	Children	4.5 (3.4–5.8)	1		1	
	Adolescents	14.5 (11.9–17.6)	2.98 (2.10–4.22)	**<0.001**	3.93 (2.67–5.78)	**<0.001**
Gender	Boy	7.5 (6.0–9.3)	1		1	
	Girl	9.0 (7.2–11.3)	1.18 (0.85–1.62)	0.325	1.01 (0.72–1.41)	0.949
Time on first-line ART	<24 months	9.2 (6.9–12.5)	1		1	
	24–48 months	7.7 (6.0–9.8)	0.75 (0.50–1.12)	0.159	0.64 (0.42–0.96)	**0.032**
	>48 months	8.2 (6.1–10.9)	0.72 (0.46–1.11)	0.136	0.53 (0.33–0.85)	**0.008**
Calendar year (continuous)			0.97 (0.87–1.08)	0.607	0.99 (0.89–1.11)	0.896
Viral load at switch (copies/ml)	<1000	11.7 (4.9–28.2)	1		1	
	1000–10,000	5.2 (2.9–9.4)	0.52 (0.18–1.54)	0.240	0.49 (0.16–1.47)	0.203
	10,000–100,000	8.6 (6.1–12.1)	0.81 (0.31–2.13)	0.675	0.92 (0.34–2.51)	0.877
	>100.000	11.0 (7.8–15.5)	0.94 (0.36–2.48)	0.902	1.18 (0.42–3.33)	0.757
	Not available	7.6 (6.1–9.6)				
CD4 status at switch^a^	Normal	5.4 (3.4–8.6)	1			
	Diminished	9.8 (7.2–13.3)	1.52 (0.87–2.65)	0.144	1.08 (0.60–1.97)	0.791
	Immunodeficient	14.9 (10.0–22.2)	2.19 (1.12–4.26)	**0.022**	1.51 (0.72–3.18)	0.280
	Not available	7.3 (5.8–9.2)				

Virologic failure is defined as two consecutive viral load results >1000 copies/ml after at least six months of second-line ART. Cox regression model is stratified by number of VL results per child and by study cohort. Age, calendar year, viral load and CD4 status are at moment of treatment switch. ^a^Normal: CD4 count >500 or CD4% >25; diminished: CD4 count 100–500 or CD4% 10–25%; immunodeficient: CD4 count <100 or CD4% <10%. Status is based on CD4% for children younger than five years and on CD4 count for children five years or older. 95% CI: 95% confidence interval; ART: antiretroviral treatment.

## Discussion

The rate of virologic failure among children on second-line PI-based ART after failure of first-line NNRTI-based ART was 8.2 per 100 person-years, with 16.4% of children experiencing virologic failure within 24 months. Adolescents were almost four times more likely to experience virologic failure on second-line ART compared to younger children.

In the first two years of PI-based second-line ART, 16% of children experienced virologic failure. In a secondary analysis using a single VL measurement to define virologic failure, 25% failed. These outcomes are better than the pooled estimates of 60–75% of children achieving viral suppression in the first two years of first-line ART [11], which might reflect the higher potency and the higher “forgiveness of non-adherence” attributed to PIs compared to NNRTIs (mainly used in first-line regimens within LMIC). Moreover, a child’s or caregiver’s improved motivation to adhere to treatment after failure of first-line treatment might also play a role. Finally, the risk of pretreatment HIV drug resistance (HIVDR) towards NNRTIs is typically higher than resistance towards PIs, especially after exposure to drugs for the prevention of mother-to-child transmission [28,29].

Our results are comparable to outcomes of adults on second-line ART in LMIC. In a study among adults in 12 Asian countries, the failure rate was 8.8 per 100 patient-years [20]. A large cohort study in six African countries among adults on second-line ART found that 15% experienced virologic failure after two years [30]. When only looking at the subgroup of younger children in our study, outcomes are even better than adult results, as the failure rate was 4.5 per 100 patient-years and 9.1% failed after two years. This again underscores the necessity for increasing virologic monitoring as children grow older.

Adolescent age was the strongest predictor of virologic failure. This finding was robust across several sensitivity analyses. This result is in line with previous paediatric studies which also identified adolescents to be at increased risk of treatment failure on first- and second-line ART [17,18,31,32]. Studies on treatment outcomes of adolescents living with HIV in LMIC are scarce, but the available data are worrisome. In sub-Saharan Africa, HIV is the leading cause of mortality in adolescents [33]. While the number of AIDS-related deaths worldwide decreased by 24% between 2004 and 2011, mortality increased by 50% among adolescents over the same time period [13]. The observation that perinatally infected children are now surviving into adolescence due to the large-scale introduction of ART is an enormous achievement. However, the poor treatment outcomes of these children once they reach adolescence call for increased attention for this age group, not only to prevent HIV-related morbidity and mortality but also to decrease the risk of HIV transmission in sexually active adolescents. Treatment of adolescents is known to be challenging because of physical, psychological and social changes in an adolescent’s life and the transition from paediatric into adult HIV care [12,14,34,35]. Strategies to retain adolescents in care and to improve treatment adherence need to be explored further and might include adherence clubs, mobile health technologies, support in dealing with stigma, weekends-off regimens, conditional cash incentives and training in life skills and problem-solving [12,13,36,37].

Children who spent more time on first-line ART had a lower risk of virological failure. This could be explained by the assumption that children who had been on first-line ART for a long time are possibly more adherent to treatment. Therefore, the risk of second-line failure is lower for these children. The association did not reach significance in sensitivity analyses.

It is reassuring to note that children who fail first-line NNRTI-based treatment still have a good chance of re-suppressing on LPV/r-based second-line ART. However, given that WHO guidelines have recommended PI regimens as first line for young children since 2013 [23], it is concerning that hardly any data exist on outcomes of children who have failed PI-based first line and need to be switched to second-line ART. In our literature search, we identified reports of only 30 children on second-line treatment after failure of a PI-based first-line regimen, indicating that this is a profoundly understudied topic.

The most recent WHO guidelines recommend the use of a raltegravir-based regimen as a second-line option after failure of first-line LPV/r-based ART in children younger than three years and the use of either raltegravir or efavirenz in children three years or older. Children failing second-line PI-based ART are advised to start raltegravir, darunavir or dolutegravir plus two nucleoside reverse transcriptase inhibitors as a salvage regimen [3]. However, despite recent Medicines Patent Pool licensing agreements allowing royalty-free manufacturing for sale in LMIC [38], these drugs are hardly available in programmatic settings in LMIC [23,39–41]. For children failing PI-based treatment (either as a first line for young children or as a second line for older children), treatment options are therefore still very limited.

Unfortunately, most cohorts included in this analysis did not perform genotypic HIVDR testing in children failing second-line ART. To our knowledge, only two published cohort studies in Asia described HIVDR patterns in a cohort of children on second-line ART. These reported relatively low rates of PI resistance of 11.3% and 8.0% in children failing second line [18,32]. In a cohort of 64 children on second line in Uganda, no PI mutations were detected after failure [42]. Studies among adults on second-line ART confirm that failure is driven mainly by non-adherence and not by PI resistance. Nevertheless, if adherence is suboptimal over longer periods of time, PI mutations will eventually develop [43–45].

One of the major strengths of this study is the fact that this is the largest analysis of paediatric outcomes on second-line ART in LMIC to date, combining data from both Asia and sub-Saharan Africa. Our results confirm the findings of previous smaller studies, showing that failure rates are relatively favourable for children, but worryingly high for adolescents. Our study also has some potential limitations. First, this was a multicentre analysis in which treatment protocols and the availability of data differed by study site. Therefore, the data used in this analysis are heterogeneous, and this heterogeneity should be kept in mind when interpreting the results. We attempted to minimize the influence of this heterogeneity by clustering our analysis by study cohort, by conducting a secondary analysis of the incidence of virological failure and by conducting various sensitivity analyses.

Second, by definition, a child could only be classified as experiencing failure when a VL result was available. A child with more VL results, therefore, had more chance to be classified as experiencing virologic failure, creating a bias towards more virological failure at study sites with more frequent VL monitoring. To correct for this, we stratified our Cox model for different number of VL measurements per child. It is striking to note that, even in controlled research settings, important clinical data such as VL and CD4 count are missing for a large proportion of children. To improve clinical outcomes of children on ART, intensified treatment monitoring is essential.

Third, some potentially relevant variables were not included in our analysis such as adherence, HIVDR, ART regimen and clinical status because not all studies had collected these data or because data were too heterogeneous to include.

Fourth, some cohorts we approached for this analysis either did not reply to our request or could not share their data. This might have created a bias in our analysis.

Finally, our study concerns children on second-line PI-based ART after failure of an NNRTI-based first-line regimen, which is not currently the recommended ART sequence for young children. However, since PI-based first-line treatment has not yet been widely implemented in LMIC and given that many children are only diagnosed with HIV after the age of three years, our data are applicable to the large number of HIV-infected children who will still receive NNRTI-based first-line ART. If we would have included children in our analysis on other second-line regimens, such as NNRTI-based second-line ART, the rate of virologic suppression we found might have been lower, given the poor results of NNRTI-based second-line ART found in studies in South Africa [46,47].

## Conclusions

In conclusion, this multicentre analysis shows that treatment outcomes of children on second-line PI-based ART are encouraging with 16% experiencing virologic failure in the first two years. However, for the 16% who fail second-line ART, integrase inhibitors, which are currently not available in LMIC, are needed to construct salvage regimens. Data on second-line treatment outcomes after failure of a PI-based regimen are hardly available and therefore urgently needed. The poor outcomes of adolescents compared to younger children underscore the difficulties of adolescent HIV care and call for increased attention for this vulnerable population as they transition into adulthood.
